# β-Thujaplicin Mobilizes Renal Tubular Iron to Alleviate Diabetic Kidney Disease

**DOI:** 10.1080/0886022X.2026.2657666

**Published:** 2026-04-29

**Authors:** Fengning Yang, Kewu Wang, Jiayi Chu, Shan Huang, Qian Wu, Xiufang Xu, Wenbo Xiao

**Affiliations:** ^a^ School of Medical Imaging, Hangzhou Medical College, Hangzhou, China; ^b^ Department of Radiology, The First Affiliated Hospital of Zhejiang University, Hangzhou, China; ^c^ Department of Radiology, Center of Regenerative and Aging Medicine, the Fourth Affiliated Hospital of School of Medicine, and International School of Medicine, International Institutes of Medicine, Zhejiang University, Yiwu, China

**Keywords:** Diabetic kidney disease, β-Thujaplicin, iron, renal fibrosis

## Abstract

Diabetic kidney disease (DKD) is a major complication of diabetes mellitus. Iron metabolism is implicated in the pathogenesis of DKD; however, its mechanistic basis and therapeutic implications remain incompletely explored. Here, we employed unilateral nephrectomy combined with streptozotocin-induced DKD mice to identify distinct renal iron metabolic profiles in DKD versus controls. Notably, although total renal iron content remained unchanged, pronounced iron deposition was detected in proximal renal tubules of DKD mice compared with controls. This aberrant iron deposition correlated with elevated fibrotic markers, indicating a potential mechanistic link between iron dysregulation and renal fibrosis in DKD. Importantly, the iron ionophore β-Thujaplicin (hinokitiol) effectively mobilized tubular iron and significantly attenuated renal fibrosis. Transcriptome analysis revealed that TGF-β signaling, a key fibrotic pathway, was inhibited by β-Thujaplicin. To further elucidate the mechanism of β-Thujaplicin mitigating renal fibrosis, HK-2 cells were exposed to a combination of high glucose, palmitate, and TGF-β1. The results demonstrated that THU alleviated fibrosis in HK-2 cells by reducing oxidative stress, decreasing intracellular iron levels, and inhibiting the TGF-β signaling pathway. Collectively, our findings establish that iron metabolic reprogramming drives DKD progression, and pharmacological targeting of tubular iron deposition *via* iron ionophores represents a promising strategy against renal fibrosis in DKD and related nephropathies.

## Introduction

Diabetic kidney disease (DKD) is a major complication of the diabetic milieu, affecting approximately half of patients with type 2 diabetes and one-third of those with type 1 diabetes [1]. This condition is primarily characterized by glomerular capillary lesions, driving progressive loss of renal function and manifesting as nephrotic syndrome, diffuse glomerulosclerosis, and glomerular hypertrophy [2].

The pathogenesis of DKD involves multifactorial processes, including hyperglycemia, aberrant renal hemodynamics, oxidative stress, inflammatory responses, and genetic factors [3]. In healthy kidneys, iron is tightly regulated by proteins like transferrin receptor 1 (TFR1) and ferritin, maintaining homeostasis. In DKD, this balance is disrupted, leading to iron accumulation in proximal tubules [4,5]. The hypoxic microenvironment in DKD kidneys promotes reactive oxygen species (ROS) generation [6], which triggers lipid peroxidation and culminates in ferroptosis—an iron-dependent programmed cell death pathway [7]. Notably, the kidney represents a prime target organ for ferroptosis, as evidenced by kidney damage upon genetic deletion of glutathione peroxidase 4 (GPX4), a master ferroptosis suppressor, in mice [8]. Consequently, ferroptosis has emerged as a pivotal mechanism underlying acute kidney injury [9], chronic kidney disease (CKD) [10], and DKD [11].

Targeting iron metabolism and ferroptosis pathways constitutes a promising therapeutic avenue for DKD and related fibrotic nephropathies. Iron chelators including deferoxamine (DFO) and deferiprone (DFP) demonstrate renoprotective effects in experimental kidney disease [12,13]. Nevertheless, their iron-restrictive action may exacerbate anemia—a prevalent complication in CKD and DKD [14,15]. Hence, iron remobilization strategies present a viable alternative to circumvent this limitation. Unlike SGLT2 inhibitors (used for treatment of anemia in DKD), which target hemodynamic and inflammatory pathways in DKD [16], β-Thujaplicin (hinokitiol) addresses iron dysregulation, offering a complementary therapeutic approach.

Herein, we established a DKD mouse model *via* unilateral nephrectomy plus streptozotocin-induced type 1 diabetes, and complemented these *in vivo* studies with *in vitro* experiments in which HK‑2 cells were exposed to high glucose, palmitate, and TGF‑β1. We identified proximal tubular iron accumulation in these models, and found a curative effect of the metal ionophore β-Thujaplicin which at least partially depended on its iron mobilization capacity.

## Materials and methods

### Animals

Male C57BL/6 mice aged 6–8 weeks were obtained from GemPharmatech. All procedures were approved by the Institutional Animal Care and Use Committee of Zhejiang University (Protocol No. ZJU20230141) and conducted in accordance with the National Institutes of Health Guidelines for Animal Care. Mice were randomly assigned to experimental groups and housed in individually ventilated cages under controlled conditions (22–24 °C, 12 h light/dark cycle) with *ad libitum* access to distilled water and standard chow diet (XTI01FZ-010, Jiangsu Xietong Pharmaceutical Bio-Engineering Co., Ltd.). Adult mice aged 6–28 weeks were utilized throughout the study.

### Diabetic kidney disease model induction

The DKD model was established through unilateral nephrectomy to increase renal metabolic burden, followed by streptozotocin (STZ) administration as previously described [17,18]. Briefly, 6-8-week-old mice were anesthetized with 1% sodium pentobarbital (8 μL/g body weight, i.p.). After shaving the dorsolateral left flank, the surgical site was disinfected with 10% povidone-iodine. A 5-mm vertical incision parallel to the spine was made through skin and muscle layers. The left kidney was gently extruded, and the renal vasculature and ureter were ligated prior to excision. Incisions were sutured, and mice recovered on heating pads until ambulatory.

Following a 2-week recovery period, mice received intraperitoneal injections of STZ (55 mg/kg/day) for five consecutive days. Diabetic status was monitored biweekly *via* tail vein blood glucose measurements, body weight recording, and rectal temperature assessment. At week 20 post-STZ induction, mice were euthanized. β-Thujaplicin (THU; Sigma-Aldrich #469521) was dissolved in PBS containing 5% ethanol. From week 19 to 20, treatment groups received daily intraperitoneal injections (5 consecutive days/week) of either β-Thujaplicin (10 mg/kg) or vehicle control (PBS/5% ethanol), totaling 10 administrations per mouse.

At week 20 post-STZ induction, all mice (body weight <200 g) received an intraperitoneal injection of 300 μL sodium pentobarbital (10 mg/mL) to induce deep anesthesia, followed immediately by cervical dislocation. Death was confirmed by cessation of heartbeat and absence of reflexes to a firm toe pinch. All euthanasia procedures followed the AVMA Guidelines for the Euthanasia of Animals (2020 Edition).

### Grouping specification

VEC+CON: Following unilateral nephrectomy, this group of mice received only the vehicle administered in five consecutive injections over one week. After 16 weeks, they received vehicle (PBS containing 5% absolute ethanol) injections five times per week for two consecutive weeks.

VEC+THU: Following unilateral nephrectomy, this group of mice received only the vehicle administered in five consecutive injections over one week. After 16 weeks, they received β-Thujaplicin (dissolved in PBS containing 5% absolute ethanol) injections five times per week for two consecutive weeks.

STZ+CON: Following unilateral nephrectomy, this group of mice received only the STZ administered in five consecutive injections over one week. After 16 weeks, they received vehicle (PBS containing 5% absolute ethanol) injections five times per week for two consecutive weeks.

STZ+THU: Following unilateral nephrectomy, this group of mice received only the STZ administered in five consecutive injections over one week. After 16 weeks, they received β-Thujaplicin (dissolved in PBS containing 5% absolute ethanol) injections five times per week for two consecutive weeks.

*The groups described above correspond to* Figure 2C-J, Figure 3A-H*, Supplementary Figure 2C and D, and Supplementary Figure 3C-F.*

HP group: HK-2 cells treated with DMEM medium containing 30 mM glucose and 300 μM palmitic acid for 24 h.

HPT group: HK‑2 cells treated with DMEM medium containing 30 mM glucose, 300 μM palmitic acid, and 10 ng/mL TGF‑β1 for 24 h.

*These treatment conditions correspond to* Figure 4.

### Cell Culture

The human renal proximal tubular epithelial cell line HK-2 (Catalog #PWE-HU091) and murine glomerular mesangial cell line SV40-MES-13 (Catalog #PWE-MU033) were obtained from Meilun Biotechnology Co., Ltd. Both cell lines were maintained at 37 °C under a 5% CO_2_ atmosphere. SV40-MES-13 cells were cultured in DMEM/F12 medium (BasalMedia #L310KJ) supplemented with 5% fetal bovine serum (FBS; Meilun #PWL001) and 1% penicillin-streptomycin (BasalMedia #S110JV), while HK-2 cells were grown in DMEM medium (BasalMedia #L170KJ) containing identical concentrations of FBS and penicillin-streptomycin.

To investigate the effects of β-Thujaplicin on renal tubular cells under diabetic conditions, HK-2 cells were seeded in 12-well plates at a density of 1 × 10^5 cells per well and allowed to adhere for 12 h under standard culture conditions. Subsequently, the medium was replaced with low-glucose DMEM without fetal bovine serum (FBS) for serum starvation over 24 h. Cells were then exposed to complete DMEM medium containing 30 mM glucose, 300 μM sodium palmitate, and 10 ng/mL TGF-β1 for 24 h.

### Cell viability assay (CCK-8)

HK-2 or SV40-MES-13 cells were seeded in triplicate in 96-well plates at 10,000 cells/well. At 90-95% confluency, cells were treated with graded concentrations of β-Thujaplicin in their respective culture media (as specified in the **Cell Culture** section) for 24 h. After treatment, medium was replaced with fresh medium containing 10% Cell Counting Kit-8 (CCK-8) reagent (MA0218-2; Meilunbio). Following 1 h incubation at 37 °C with 5% CO_2_, absorbance was measured at 450 nm using a microplate reader. The same method was employed to assess the cytotoxic effect of PA on HK-2 cells. Cell viability was normalized to the control group (100%), and IC_50_ values were calculated using GraphPad Prism 9.

### Serology

Mice were euthanized and blood was collected *via* cardiac puncture. Serum levels of urea (BUN), creatinine, aspartate aminotransferase (AST), alanine aminotransferase (ALT), and lactate dehydrogenase (LDH) were measured by Haoke Biotechnology Co., Ltd. (China).

### Histology and immunohistochemistry

Mice underwent transcardial perfusion with 10 mL PBS *via* the right ventricle. Kidneys were harvested, fixed in 10% formalin, embedded in paraffin, and sectioned at 3 μm thickness. Tissue sections were stained with hematoxylin and eosin (H&E), Masson’s trichrome, periodic acid-Schiff (PAS), Prussian blue, or DAB-enhanced Perl’s Prussian blue staining.

Renal tubular injury was scored on H&E-stained cortical sections using an established system [19]. Ten random cortical fields (400× magnification) were evaluated. Histological scoring (0–4 scale) assessed four parameters: tubular dilation, brush border loss, tubular necrosis, and neutrophil infiltration. Scoring criteria: 0 = normal morphology; 1 = mild injury (<25% involvement); 2 = moderate (26-50%); 3 = severe (51-75%); 4 = extensive damage (>75%).

For immunohistochemistry, paraffin sections were dewaxed in xylene I/II (12 min each), rehydrated through graded ethanol (100%, 95%, 85%; 6 min each), and rinsed in 1× PBS. Antigen retrieval used preheated 20× Tris-EDTA buffer (pH 9.0) with microwave irradiation (8 min boiling, 8 min standing, 7 min medium-low power). After cooling and PBS washing (3 × 5 min), endogenous peroxidase was blocked with 3% H_2_O_2_ (25 min, dark). Sections were outlined with a hydrophobic barrier pen and blocked with 3% BSA (30 min). Primary antibodies (Fth1 1:100, Fibronectin 1:200, α-Sma 1:200) were applied overnight at 4 °C. After PBS washing, HRP-conjugated secondary antibodies were incubated (50 min, RT). DAB substrate (freshly prepared) was applied under microscopic monitoring, followed by hematoxylin counterstaining. Sections were dehydrated through graded ethanol/xylene, mounted with neutral resin, and scanned using 3DHistech SCAN II. Images were analyzed with SlideViewer software. Immunohistochemical quantification used ImageJ, while other parameters were analyzed with Image-Pro Plus 6.0.

### Western blotting

Tissue lysates were prepared in 2× SDS sample buffer containing protease inhibitor cocktail (Roche, 11873580001). Total protein concentration was determined by measuring absorbance at 280 nm (NanoDrop X). Equal protein amounts were resolved on precast 4-20% gradient SDS-PAGE gels (ACE, ET15420LGel) and electrotransferred to 0.2 μm polyvinylidene fluoride (PVDF) membranes.

Membranes were blocked with 5% nonfat milk in TBST for 2 h, washed, and probed with primary antibodies overnight at 4 °C. Antibodies included: anti-Fth1 (ab75973, Abcam), anti-α-SMA (14-9760-82, Thermo Fisher), anti-Fibronectin (ab2413, Abcam), anti-HO-1 (#43966, CST), anti-HO-2 (14817-1-AP, Proteintech), anti-TFR1 (13–6800, Thermo Fisher), anti-p-Smad2/3(#8828, CST), and Smad2/3(#5678S, CST).

After TBST washes (3 × 10 min), membranes were incubated with HRP-conjugated secondary antibodies: goat anti-rabbit (AS014, Abclonal) and goat anti-mouse (AS003, Abclonal). Following additional TBST washes (3 × 10 min), chemiluminescent signals were developed using ECL substrate (32106, Thermo Fisher) and captured on a Bio-Rad ChemiDoc Touch system. Band density quantification was performed with ImageJ using non-saturated exposures.

### Urinary albumin-to-creatinine ratio (ACR)

Urinary microalbumin was quantified using ELISA (E-EL-M0792, Elabscience) according to manufacturer specifications. Urinary creatinine levels were assessed with a Creatinine Assay Kit (C011-2-1; Njjcbio). The ACR was calculated as microalbumin concentration divided by creatinine concentration.

### RNA sequencing

Renal tissues were snap-frozen in liquid nitrogen and submitted to MajorBio for RNA extraction and sequencing. Total RNA was isolated using TRIzol Reagent. RNA integrity was assessed with an Agilent 5300 Bioanalyzer and quantified *via* NanoDrop 2000. High-quality samples (OD260/280 = 1.8–2.2, OD260/230 ≥ 2.0, RIN ≥ 6.5, 28S:18S ≥ 1.0) were selected for library construction. Strand-specific mRNA libraries were prepared using the Illumina Stranded mRNA Prep Ligation Kit with 1 μg total RNA. cDNA libraries were size-selected (300 bp) on 2% Low Range Ultra Agarose gels, PCR-amplified, and quantified with Qubit 4.0. Paired-end sequencing (2 × 150 bp) was performed on an Illumina NovaSeq X Plus platform. Raw reads were processed using standardized bioinformatics pipelines.

Differentially expressed genes (DEGs) between streptozotocin (STZ)-treated and vehicle (VEC) groups, or STZ+THU versus STZ groups, were identified based on transcripts per million (TPM). Transcript abundance was quantified with RSEM [20], and differential expression analysis employed DESeq2 or DEGseq. DEGs meeting |log_2_ FC| ≥ 1 with FDR ≤ 0.05 (DESeq2) or FDR ≤ 0.001 (DEGseq) were considered significant. Functional enrichment analysis (Gene Ontology [GO] and Kyoto Encyclopedia of Genes and Genomes [KEGG]) was performed using GOATools and KOBAS [21], with significance defined as Bonferroni-adjusted *p* ≤ 0.05 using the whole transcriptome as background.

### Inductively coupled plasma mass spectrometry (ICP-MS)

Kidney samples were digested with 400 μL nitric acid and 100 μL hydrogen peroxide (H_2_O_2_), followed by 10-min incubation at room temperature. Microwave-assisted digestion was then conducted at 90 °C for 1 h. Digested samples were diluted to 10 mL with deionized water, vortexed for 30 s, and analyzed by ICP-MS.

Quantification utilized internal standardization. Calibration curves were generated by plotting known metal standard concentrations (x-axis) against analyte-to-internal standard signal ratios (y-axis). Renal metal concentrations were derived from curve-fitting equations.

### Iron quantification

Renal non-heme iron content was measured colorimetrically [22]. Briefly, around 0.1 g tissue was homogenized in 1 mL digestion solution (containing 350 mL concentrated hydrochloric acid, 100 g trichloroacetic acid, and 650 mL ddH_2_O) and incubated at 65 °C for 50 h in a drying oven with periodic agitation (3 × 15 min intervals). After centrifugation, supernatants were adjusted to 1.5 mL with fresh digestion solution and vortexed. Samples, iron standards, and blanks were dispensed into 96-well plates, reacted with chromogenic reagent, and absorbance at 535 nm was measured. Iron concentrations were calculated against standard curves.

### Flow cytometry

#### ROS detection

After reagent treatment, HK-2 cells were washed once with 1× PBS. Then, 500 μL of 2 μM DCFH-DA in 1 × PBS (#S0033S, Beyotime Biotechnology) was added to each well, and the cells were incubated at 37 °C for 30 min. After washing three times with 1× PBS, the cells were digested with 200 μL of trypsin for 2 min, and the digestion was stopped by adding low-glucose DMEM complete medium. The cell suspension was centrifuged at 400 g, washed once with 1% FACS buffer, and stained with aqua viability dye for 10 min in the dark. After another wash with 1% FACS buffer (1 × PBS with 2% fetal bovine serum), the cells were filtered through a 350-mesh strainer into flow cytometry tubes and analyzed on the flow cytometer (BD Fortessa).

### FerroOrange staining

After reagent treatment, HK-2 cells were washed once with 1× PBS. Then, 500 μL of 2 μM FerroOrange in 1 × PBS was added to each well, and the cells were incubated at 37 °C for 30 min. After washing three times with 1× PBS, the cells were digested with 200 μL of trypsin for 2 min, and the digestion was stopped by adding low-glucose DMEM complete medium. The cell suspension was centrifuged at 400 g, washed once with 1% FACS buffer, and stained with aqua viability dye for 10 min in the dark. After another wash with 1% FACS buffer, the cells were filtered through a 350-mesh strainer into flow cytometry tubes and analyzed on the flow cytometer (BD Fortessa).

### Statistical analysis

All statistical analyses were performed using GraphPad Prism 10 software with quantitative data presented as mean ± standard deviation (SD) unless otherwise specified. For comparisons between two independent groups, unpaired Student’s t-tests with Welch’s correction or Mann-Whitney U tests were employed as appropriate based on distribution normality and variance homogeneity; paired t-tests were applied for within-group longitudinal comparisons. Analyses involving three or more groups utilized one-way ANOVA or two-way ANOVA with appropriate *post hoc* testing. Comprehensive statistical parameters including exact *P*-values, specific test types, degrees of freedom, and effect sizes are fully documented in corresponding figure legends. Post hoc power analyses were performed using G*Power 3.1.9.7 to assess sample size adequacy. These statistical power values are listed in the *Supplementary Table S1_Statistical reports*.

## Results

### Iron is redistributed in the kidneys from diabetic mice

**Figure 1.**
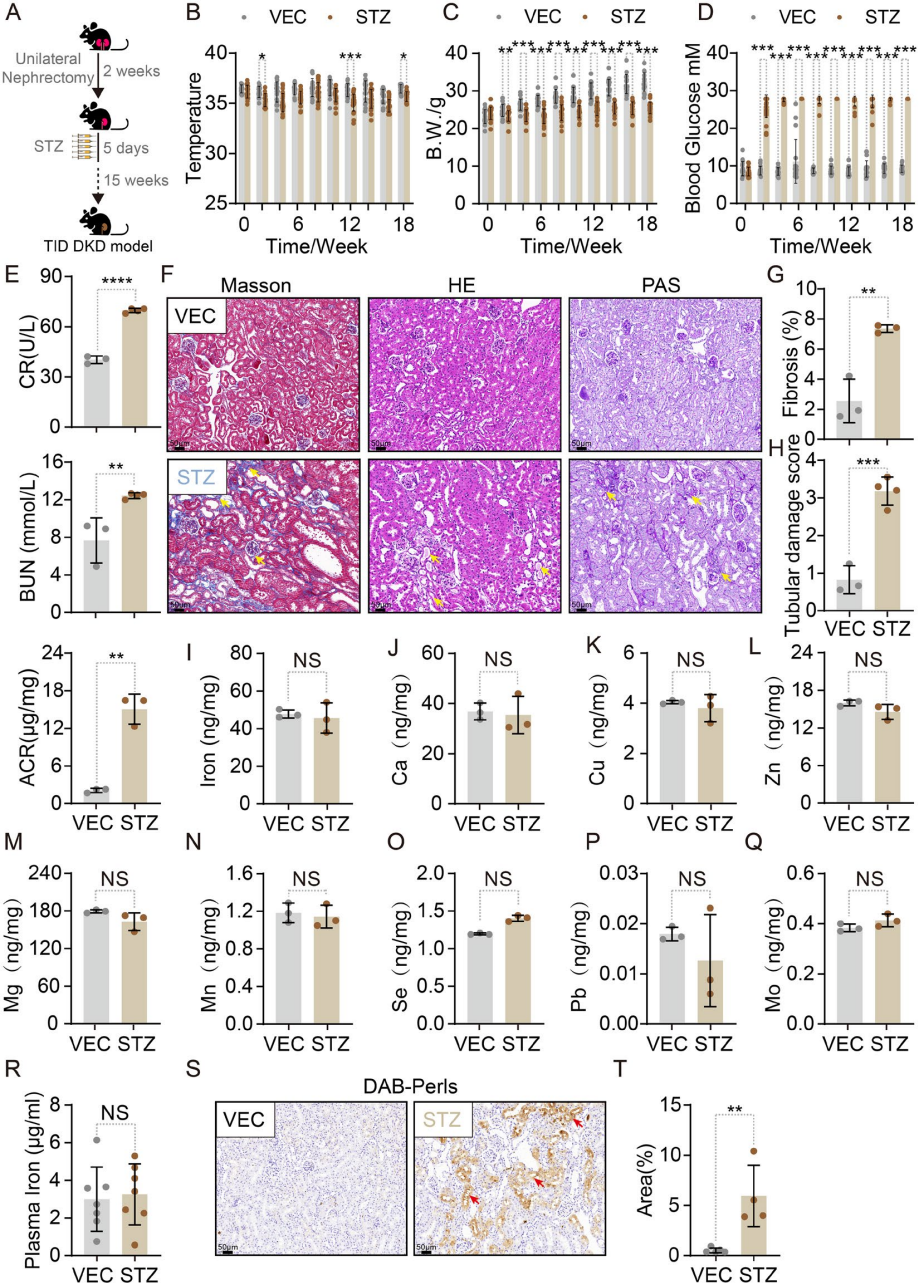
DKD mice exhibit abnormal renal iron distribution. (A) Schematic of diabetic kidney disease (DKD) model induction. (B-D) Body temperature, weight, and blood glucose dynamics (0–18 weeks; VEC: *n* = 18; STZ: *n* = 23). (E) Serum creatinine (CR), blood urea nitrogen (BUN), and albumin-to-creatinine ratio (ACR) at 18 weeks (VEC: *n* = 3; STZ: *n* = 4). (F) Representative Masson’s trichrome, H&E, and PAS staining (VEC: *n* = 3; STZ: *n* = 4). (G) Collagen deposition quantification (*n* = 3). (H) Renal tubular injury scores (VEC: *n* = 3; STZ: *n* = 4). (I) Renal iron content by ICP-MS (*n* = 3). (J-Q) Renal calcium (Ca), copper (Cu), zinc (Zn), magnesium (Mg), manganese (Mn), selenium (Se), lead (Pb), and molybdenum (Mo) levels by ICP-MS (*n* = 3). (R) Plasma iron concentrations (*n* = 7). (S) DAB-enhanced Perls Prussian blue staining (VEC: *n* = 5; STZ: *n* = 4). (T) Iron deposition quantification (VEC: *n* = 5; STZ: *n* = 4). Data sources: B-D pooled from 3 experiments; E, G-Q, T from single experiment; R pooled from 2 experiments; F and S show representative images. Data presented as mean ± SD. Statistics: Two-way ANOVA (B-D); Student’s t-test (E, G-Q, T). Significance: NS: *p* > 0.05, *: *p* < 0.05, **: *p* < 0.01, ***: *p* < 0.001, ****: *p* < 0.0001.

To establish a diabetic kidney disease (DKD) model, unilateral nephrectomy was performed in 6–8-week-old male C57BL/6 mice (a strain resistant to conventional DKD induction) followed by streptozotocin (STZ) administration (Figure 1A) [17,18]. Compared to vehicle (VEC)-treated controls, STZ-administered mice exhibited reduced body weight, hypothermia, and sustained hyperglycemia (Figure 1B-D). At 16 weeks post-induction, STZ-administered mice demonstrated elevated blood urea nitrogen (BUN), plasma creatinine, and urinary albumin-to-creatinine ratio (ACR) (Figure 1E). Masson’s trichrome staining revealed increased renal collagen deposition in STZ-administered mice (Figure 1F and G), while H&E and PAS staining confirmed exacerbated tubular injury and higher histopathological scores (Figure 1F and H). These findings validate successful DKD modeling in C57BL/6 mice.

Inductively coupled plasma mass spectrometry (ICP-MS) analysis detected no significant alterations in renal iron, zinc, copper, manganese, calcium, magnesium, or selenium levels between STZ and VEC groups (Figure 1I-Q). Similarly, circulating iron remained unchanged (Figure 1R). These data indicate preserved total renal iron content and systemic iron homeostasis in DKD.

To delineate spatial iron distribution, we employed DAB-enhanced Perls Prussian blue staining (enhanced sensitivity *vs.* conventional staining; *Supplementary Figure 1A*). STZ kidneys exhibited pronounced iron deposition specifically within proximal tubules compared to VEC controls (Figure 1S and T). This aligns with prior reports of proximal tubular enrichment in iron and mitochondria [22,23]. Collectively, these results suggest that diabetic conditions trigger intrarenal iron redistribution—predominantly to proximal tubules—rather than total renal iron accumulation.

### Iron ionophore β-thujaplicin rebalanced renal iron distribution and ameliorated DKD

**Figure 2.**
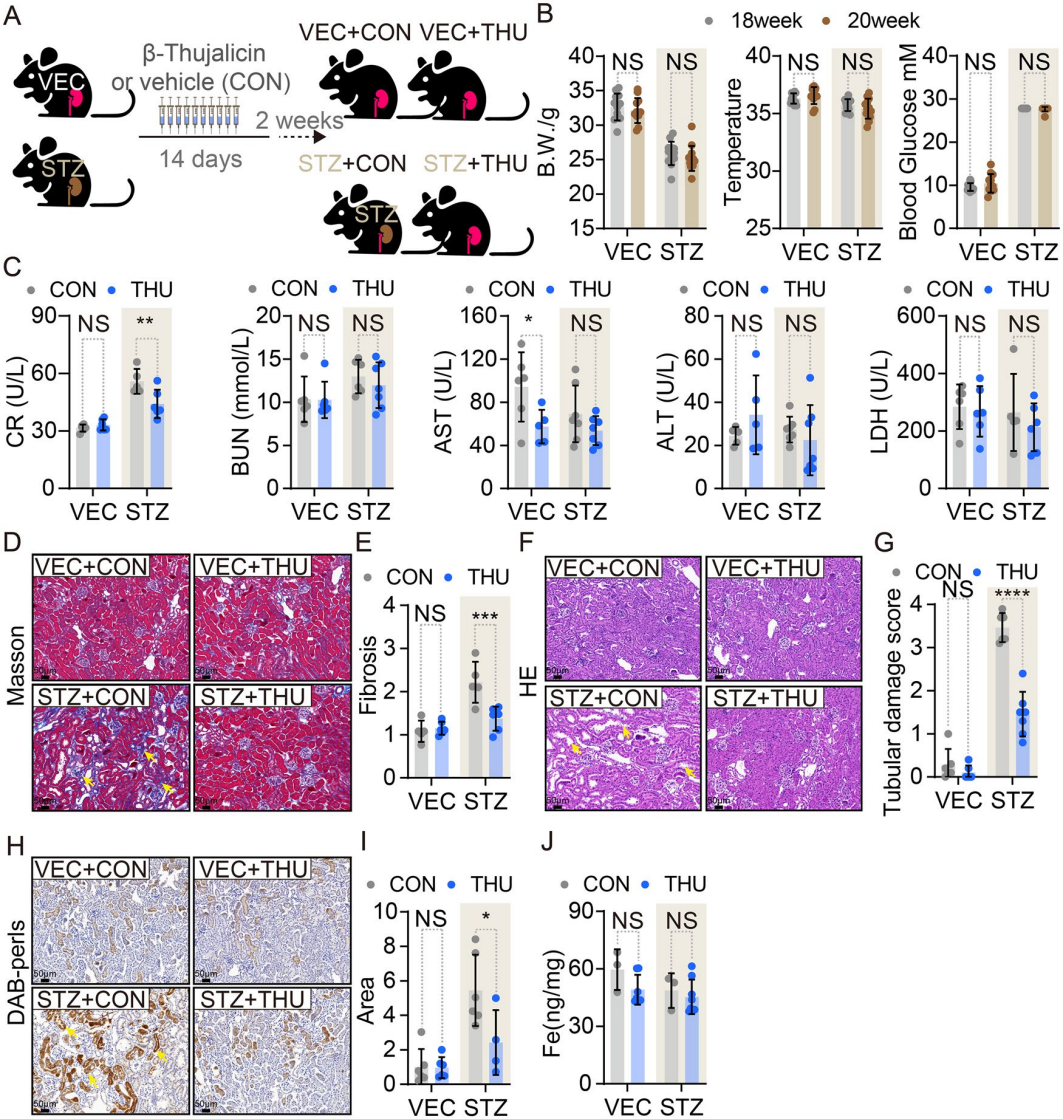
β-Thujaplicin leads to iron redistribution and alleviates fibrosis in the kidney of DKD mice. (A) Schematic diagram of β-Thujaplicin administration in DKD mice. (B) Body weight, core temperature, and blood glucose pre-/post-treatment (VEC: *n* = 12; STZ: *n* = 13). (C) CR, BUN, AST, ALT, and LDH levels (VEC+CON: *n* = 6; VEC+THU: *n* = 6; STZ+CON: *n* = 7; STZ+THU: *n* = 7). (D) Representative Masson’s trichrome-stained sections (VEC+CON: *n* = 5; VEC+THU: *n* = 6; STZ+CON: *n* = 5; STZ+THU: *n* = 6). (E) Quantified collagen deposition (VEC+CON: *n* = 5; VEC+THU: *n* = 6; STZ+CON: *n* = 5; STZ+THU: *n* = 6). (F) H&E-stained sections (VEC+CON: *n* = 6; VEC+THU: *n* = 6; STZ+CON: *n* = 6; STZ+THU: *n* = 7). (G) Tubular injury scores (VEC+CON: *n* = 6; VEC+THU: *n* = 6; STZ+CON: *n* = 6; STZ+THU: *n* = 7). (H) DAB-enhanced Perls Prussian blue staining (VEC+CON: *n* = 6; VEC+THU: *n* = 6; STZ+CON: *n* = 6; STZ+THU: *n* = 4). (I) Quantified iron deposition (VEC+CON: *n* = 6; VEC+THU: *n* = 6; STZ+CON: *n* = 6; STZ+THU: *n* = 4). (J) Renal iron content by ICP-MS (VEC+CON: *n* = 3; VEC+THU: *n* = 7; STZ+CON: *n* = 3; STZ+THU: *n* = 8). Data sources: B, C, E, G, I, J pooled from two experiments; D, F, H show representative images. Data presented as mean ± SD. Statistics: Two-way ANOVA (B, C, E, G, I, J). Significance: NS: *p* > 0.05, *: *p* < 0.05, **: *p* < 0.01, ***: *p* < 0.001, ****: *p* < 0.0001.

To investigate whether rectifying proximal tubular iron mislocalization could mitigate DKD, we employed the iron ionophore β-Thujaplicin (THU) to mobilize sequestered iron (Figure 2A). THU—a tropolone derivative previously shown to induce iron efflux in macrophages [24]—demonstrated low cytotoxicity in renal cells: EC_50_ = 59.25 μM in SV40-MES-13 mesangial cells, with no observed toxicity in HK-2 tubular epithelial cells after 24-h exposure (*Supplementary Figure 2A and B*). Based on *in vitro*-*in vivo* dose translation, 10 mg/kg was selected for *in vivo* studies. Unilateral nephrectomized mice received streptozotocin (STZ) or vehicle (VEC) to induce type 1 diabetes. At 18 weeks post-induction, THU or vehicle (CON) was administered daily for 5 consecutive days/week over two weeks (Figure 2A). THU treatment did not alter blood glucose, body weight, or rectal temperature in STZ or VEC mice (Figure 2B). At week 20, THU reduced plasma creatinine (but not BUN, ALT, AST, or LDH) in STZ+THU mice versus STZ+CON controls, while only AST decreased in VEC+THU mice (Figure 2C). Critically, Masson’s trichrome staining revealed attenuated renal collagen deposition in STZ+THU mice (Figure 2D and E). H&E and PAS staining confirmed improved renal morphology and lower injury scores in STZ+THU versus STZ+CON mice (Figure 2F-G *and Supplementary Figure 2C*), with no effects in non-diabetic groups.

DAB-enhanced Perls Prussian blue staining demonstrated reduced tubular iron accumulation in STZ+THU mice (Figure 2H and I). ICP-MS analysis indicated unchanged renal levels of iron, manganese, copper, zinc, molybdenum, calcium, selenium, or magnesium in THU-treated groups, except decreased lead in VEC+THU versus VEC+CON (Figure 2J *and Supplementary Figure 2D*). Collectively, THU confers renal protection in DKD by rebalancing tubular iron distribution without altering total renal metal homeostasis.

### β-thujaplicin partially recovered the reshaped transcriptome in the kidney from the DKD model

**Figure 3.**
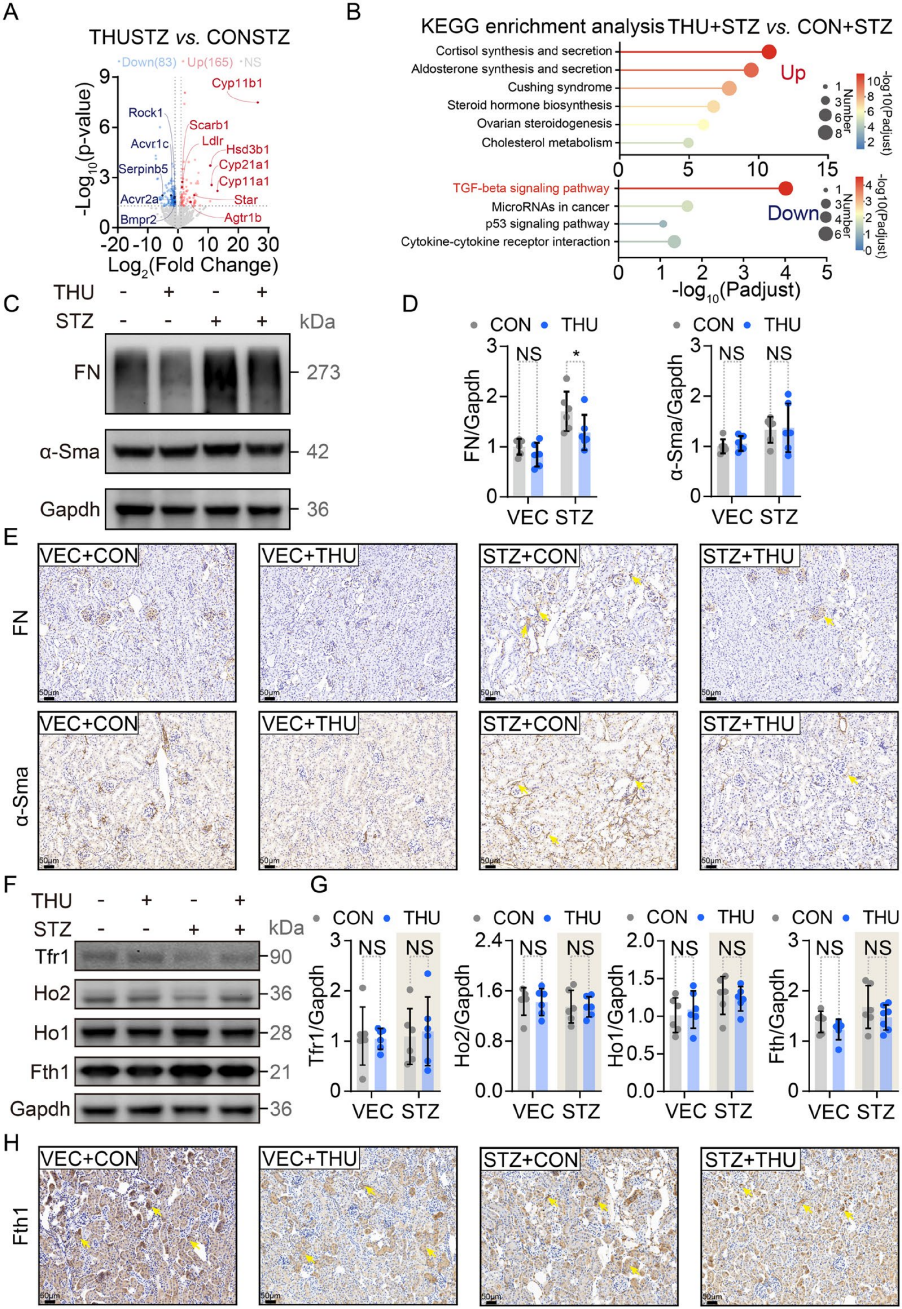
β-Thujaplicin mitigates fibrotic pathway in the kidney of DKD mice. (A) Volcano plot of differentially expressed genes (STZ+THU vs. STZ+CON; *n* = 2 biological replicates). (B) KEGG pathway enrichment analysis of up/downregulated genes (STZ+THU vs. STZ+CON; *n* = 2). (C) Representative western blots of fibronectin (FN) and α-smooth muscle actin (α-Sma) (VEC+CON: *n* = 6; VEC+THU: *n* = 6; STZ+CON: *n* = 6; STZ+THU: *n* = 6). (D) Quantification of fibronectin/α-Sma normalized to Gapdh (VEC+CON: *n* = 6; VEC+THU: *n* = 6; STZ+CON: *n* = 6; STZ+THU: *n* = 6). (E) Immunohistochemical staining of fibronectin and α-Sma (*n* = 4). (F) Western blots of transferrin receptor 1 (Tfr1), heme oxygenase-2 (Ho2), heme oxygenase-1 (Ho1), and ferritin heavy chain (Fth1) (VEC+CON: *n* = 6; VEC+THU: *n* = 6; STZ+CON: *n* = 6; STZ+THU: *n* = 6). (G) Quantification of iron-related proteins normalized to Gapdh (VEC+CON: *n* = 6; VEC+THU: *n* = 6; STZ+CON: *n* = 6; STZ+THU: *n* = 6). (H) Fth1 immunohistochemistry (*n* = 4). Data sources: A, B single experiment; D, G pooled from two experiments; C, E, F, H representative images. Data in D, G presented as mean ± SD. Statistics: Two-way ANOVA (D, G). Significance: NS: *p* > 0.05, *: *p* < 0.05.

Renal transcriptomic profiling *via* RNA sequencing across all groups identified 477 upregulated and 373 downregulated genes in STZ versus VEC mice (*Supplementary Figure 3A*), with pathway enrichment revealing augmented xenobiotic Metabolism of xenobiotics by cytochrome P450, Complement and coagulation cascades, Chemical carcinogenesis-DNA adducts, Drug metabolism-cytochrome P450, p53 signaling pathway, and suppressed Steroid hormone biosynthesis, Drug metabolism-other enzyme, Oxidative phosphorylation, Metabolism of xenobiotics by cytochrome P450, Glutathione metabolism pathway in diabetic kidneys (*Supplementary Figure 3B*). Comparative analysis of STZ+THU versus STZ+CON groups demonstrated 83 upregulated and 165 downregulated genes (Figure 3A), notably implicating TGF-β signaling as the most significantly downregulated pathway following THU treatment (Figure 3B). This transcriptional reprogramming was corroborated at the protein level: fibronectin (but not α-Sma) expression decreased in STZ+THU kidneys (Figure 3C and D), while immunohistochemistry confirmed elevated fibronectin and α-Sma in STZ+CON versus VEC+CON controls (Figure 3E *and Supplementary Figure 3C*). Critically, THU administration significantly reduced fibronectin expression with a parallel trend in α-Sma reduction in diabetic kidneys.

Importantly, iron/heme metabolism in the whole kidney remained unperturbed by THU, evidenced by stable transcription of key regulators (*Trf*, *Fth1*, *Hamp*, *Slc11a2*, *Hmox1*, *Hmox2*; *Supplementary Figure 3D*), unchanged ferroptosis-related genes (*Supplementary Figure 3E*), and consistent protein expression of Ho1, Ho2, Tfr1, and Fth1 (Figure 3F-H *and Supplementary Figure 3F*). These data suggest that THU may modulate the TGF-β pathway in the kidneys of STZ mice, thereby directly or indirectly antagonizing the development of fibrosis. However, this was not associated with alterations in iron metabolism at the whole kidney level.

### β-thujaplicin attenuates fibrosis in HG/PA/TGF-β1- exposed HK-2 cells by reducing the labile iron Pool and inhibiting oxidative stress

**Figure 4.**
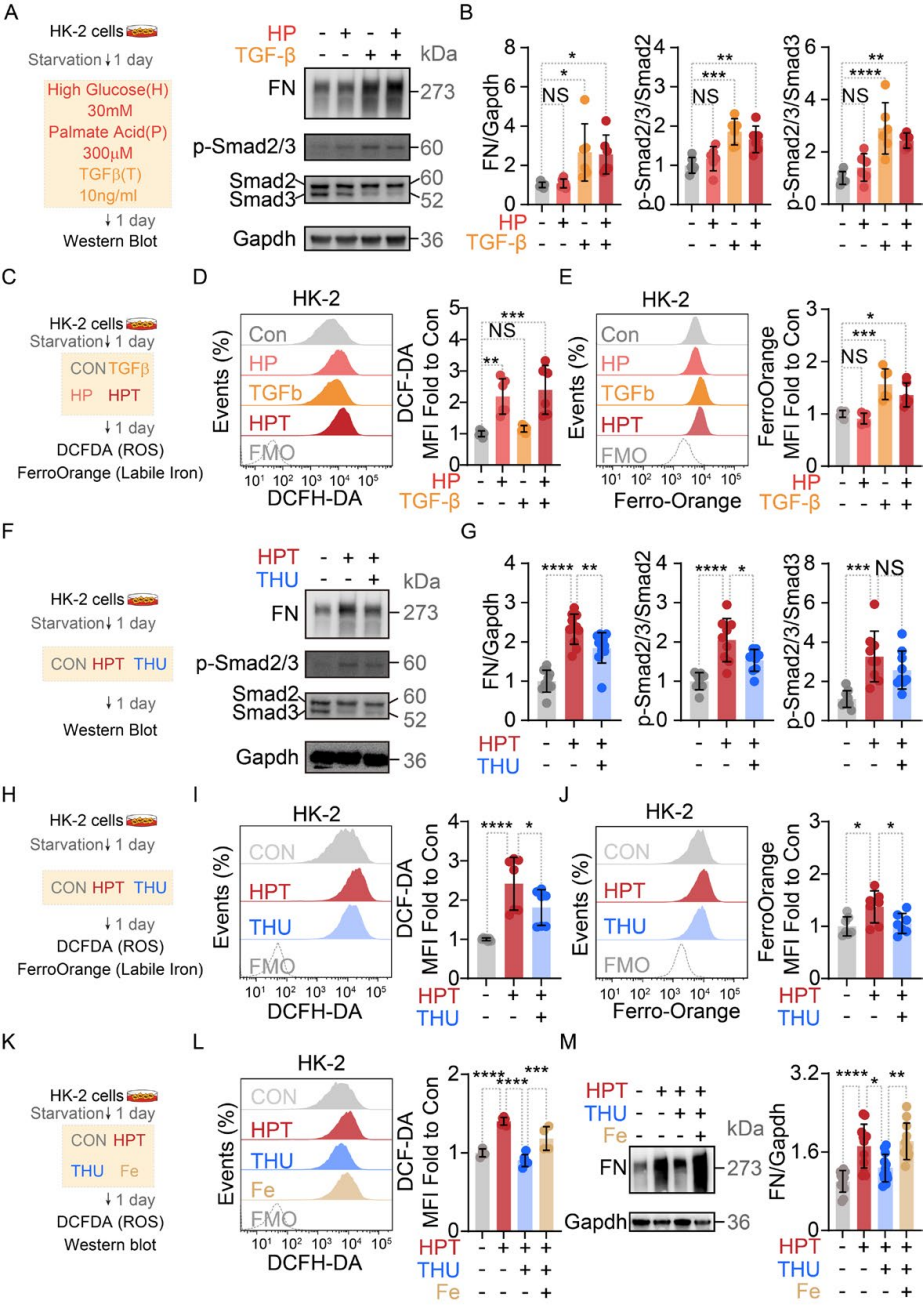
β-Thujaplicin attenuates fibrosis in HG/PA/TGF-β1-exposed HK-2 cells by reducing the labile iron pool and inhibiting oxidative stress. (A) Representative Western blot images showing protein levels of Fibronectin (FN), p-Smad2/3, Smad2, Smad3, and Gapdh in cells under different conditions. (B) Quantitative analysis of FN (normalized to Gapdh) and p-Smad2/3 (normalized to total Smad2 or Smad3). HPT treatment increased FN expression and Smad2/3 phosphorylation, indicating induced fibrosis (CON: *n* = 6; HP: *n* = 6; TGF-β: *n* = 6; HPT: *n* = 6). (C) Schematic of ROS and iron detection *via* DCFH-DA and Ferro-Orange probes in HPT-treated HK-2 cells. (D) Analysis of intracellular ROS levels using the DCFH-DA probe. Left panel: Overlay of fluorescence intensity distributions (DCF signal) from flow cytometry analysis. Right panel: Quantitative summary of mean fluorescence intensity (MFI). HPT treatment significantly increased ROS levels compared to control group (CON: *n* = 6; HP: *n* = 6; TGF-β: *n* = 6; HPT: *n* = 6). (E) Analysis of intracellular labile Fe^2+^ levels using the Ferro-Orange fluorescent probe. Left panel: Overlay of fluorescence intensity distributions (Ferro-Orange signal) from flow cytometry analysis. Right panel: Quantitative summary of mean fluorescence intensity (MFI). HPT treatment significantly increased intracellular Fe^2+^ levels compared to control groups (CON: *n* = 6; HP: *n* = 6; TGF-β: *n* = 6; HPT: *n* = 6). (F) Representative Western blot images showing protein levels of Fibronectin (FN), p-Smad2/3, Smad2, Smad3, and Gapdh in cells under different conditions. (G) Quantification of FN and p-Smad2/3 levels. THU co-treatment reduced the HPT-induced upregulation of both proteins. (H) Schematic of ROS and iron detection *via* DCFH-DA and Ferro-Orange probes in THU-treated HK-2 cells. (I) Analysis of intracellular ROS levels using the DCFH-DA probe. Left panel: Overlay of fluorescence intensity distributions (DCF signal) from flow cytometry analysis. Right panel: Quantitative summary of mean fluorescence intensity (MFI). THU treatment significantly decreased ROS levels compared to HPT groups (CON: *n* = 7; HPT: *n* = 7; THU: *n* = 7). (J) Analysis of intracellular labile Fe^2+^ levels using the Ferro-Orange fluorescent probe. Left panel: Overlay of fluorescence intensity distributions (Ferro-Orange signal) from flow cytometry analysis. Right panel: Quantitative summary of mean fluorescence intensity (MFI). THU treatment significantly decreased intracellular Fe^2+^ levels compared to HPT groups (CON: *n* = 7; HPT: *n* = 7; THU: *n* = 7). (K) Schematic of ROS and iron detection *via* DCFH-DA and Ferro-Orange probes in Fe-treated HK-2 cells. (L) Analysis of intracellular ROS levels using the DCFH-DA probe. Left panel: Overlay of fluorescence intensity distributions (DCF signal) from flow cytometry analysis. Right panel: Quantitative summary of mean fluorescence intensity (MFI). Fe treatment significantly increased ROS levels compared to THU groups (CON: *n* = 4; HPT: *n* = 6; THU: *n* = 6; Fe: *n* = 4). (M) Western blots of Fibronectin (FN), Gapdh. And quantification of FN normalized to Gapdh (CON: *n* = 12; HPT: *n* = 12; THU: *n* = 12; Fe: *n* = 12). Data sources: A-L pooled from two experiments, M pooled from four experiments. Data in B, D, E, G, I, J and L presented as mean ± SD. Statistics: One-way ANOVA (B, D, E, G, I, J, L and M). Significance: NS: *p* > 0.05, *: *p* < 0.05, **: *p* < 0.01, ***: *p* < 0.001, ****: *p* < 0.0001.

Given the protective effects of THU observed in DKD mouse models, we sought to further investigate its cellular mechanisms using HK-2 cells exposed to high glucose, palmitate, and TGF-β1 (HG/PA/TGF-β1). This exposure condition recapitulates key features of the metabolic and pro-fibrotic microenvironment of the diabetic kidney (Figure 4A) [25,26].

First, the half-maximal inhibitory concentration (IC50) of palmitate (PA) for HK-2 cells was determined to be 759.8 μM *via* CCK-8 assay. Given that PA concentrations exceeding 300 μM significantly inhibited cell viability (*Supplementary Figure 4A*), 300 μM PA was selected for subsequent experiments to balance stimulation intensity with cell activity. The protein levels of both p-Smad2/3 and fibronectin (FN) were significantly upregulated in HK-2 cells treated with either TGF-β1 alone or the combination of HG/PA and TGF-β1 (HPT) (Figure 4A,B), indicating robust activation of the TGF-β signaling pathway. HG/PA treatment significantly increased intracellular reactive oxygen species (ROS) levels determined by DCFH-DA staining, while TGF-β1 treatment specifically led to a marked elevation in intracellular iron levels, determined by FerroOrange staining (*Supplementary Figure 4B and C,* Figure 4C-E). Based on these findings, co-treatment with 300 μM PA, 30 mM glucose, and 10 ng/mL TGF-β1 was selected as the experimental condition for subsequent experiments.

To validate the effect of THU, 10 μM THU was added to HG/PA/TGF-β1-exposed HK-2 cells. Compared to the HPT group, THU treatment significantly reduced the protein levels of FN and p-Smad2/3 (Figure 4F,G), suggesting that THU can inhibit the TGF-β signaling pathway and alleviate cellular fibrosis. Concurrently, THU treatment significantly lowered the elevated intracellular ROS and labile iron pool in the model group (Figure 4H-J). These results indicated that THU can mitigate oxidative stress, iron accumulation, and fibrosis in HG/PA/TGF-β1-exposed HK-2 cells.

We hypothesized that THU might inhibit the TGF-β signaling pathway and fibrosis by reducing intracellular iron levels, thereby alleviating oxidative stress. To test this hypothesis, an iron rescue experiment was performed by adding 100 μM ferric chloride concurrently with THU treatment (Fe group) (Figure 4K). ROS levels in the Fe group were significantly higher than those in the THU group, indicating that the THU-induced reduction in ROS was reversed by iron supplementation (Figure 4L). Correspondingly, Protein level of FN in the Fe group was significantly elevated compared to the THU group, demonstrating that iron supplementation also reversed the inhibitory effect of THU on fibrosis (Figure 4M).

The above results indicate that β-Thujaplicin (THU) can attenuate HG/PA and TGF-β1-induced fibrosis in HK-2 cells. The mechanism may be related to the reduction of intracellular iron accumulation, which subsequently inhibits the ROS-mediated activation of the TGF-β/Smad signaling pathway. These data matched with our observation *in vivo*, suggesting that THU may mitigate renal fibrosis in DKD in an iron and ROS dependent mechanism.

## Discussion

Renal fibrosis represents a hallmark pathological feature of advanced kidney diseases [27,28]. Iron dysregulation and ferroptosis constitute significant contributors to renal fibrogenesis [29,30]. Consequently, substantial research has evaluated iron chelators and ferroptosis inhibitors in kidney disease models [13,31]. However, whereas iron exacerbates tissue damage in acute kidney injury [32–34], its role in chronic kidney diseases (CKD) demonstrates multifaceted complexity [35–37]. Although iron accumulates in diabetic kidney disease (DKD) patients [5], intravenous iron dextran administration proves beneficial in CKD patients [38], adenine-induced CKD models [39], and unilateral ureteral obstruction (UUO) models [40]. CKD patients frequently develop iron-deficient anemia due to impaired erythropoiesis and disrupted iron homeostasis (EPO-Erfe-hepcidin-ferroportin axis), ameliorated by parenteral iron supplementation [41]. These observations contradict therapeutic iron chelation strategies for CKD, including DKD.

We therefore propose utilizing iron ionophores—exemplified by β-Thujaplicin (THU)—to rebalance pathological iron deposition in DKD and other fibrotic nephropathies. In our study, THU did not alter total renal iron content but significantly reduced proximal tubular iron accumulation in the DKD model. Crucially, THU demonstrated therapeutic efficacy against DKD pathogenesis *in vivo* and conferred protective effects against fibrosis in HG/PA/TGF-β1-exposed HK-2 cells. These findings reveal a novel therapeutic approach for renal fibrosis: employing iron ionophores to restore iron homeostasis and attenuate fibrotic progression. As a naturally derived compound, THU exhibits anti-inflammatory, antimicrobial, and favorable safety profiles at therapeutic concentrations [42]. Further investigations should explore clinical translation of these preclinical findings for renal fibrosis management.

TGF-β signaling promotes collagen synthesis and drives fibrogenesis [43,44], our transcriptomic analysis revealed that THU suppressed TGF-β pathway in the diabetic mice. Our subsequent *in vitro* studies further supported this notion, as THU treatment blunted p-Smad2/3 phosphorylation in HG/PA/TGF-β1-exposed HK-2 cells. Our work pointed to a significant contribution of iron mobilization by THU. Nevertheless, studies have demonstrated the multiple renoprotective effects of β-Thujaplicin (hinokitiol, THU):such as mitigating endoplasmic reticulum stress in diabetic acute kidney injury (AKI) by regulating the PERK/CHOP/NF-κB axis [45], and inhibiting the TGF-β/Smad signaling pathway *via* suppression of UHRF1 expression [46]. Together, these findings suggest that THU may exert its effects through different predominant mechanisms at distinct stages of kidney injury: primarily intervening in endoplasmic reticulum stress and inflammation during the acute phase, while correcting iron metabolism dysregulation and oxidative stress during chronic fibrosis. Future research should further explore its multi-mechanistic synergistic effects throughout the dynamic progression of diabetic nephropathy.

Renal fibrosis involves multiple cell types, including parenchymal cells (renal tubular epithelial cells), immune cells (macrophages), and myofibroblasts [28]. In our DKD models, DAB-enhanced Prussian blue staining localized iron deposition predominantly within tubules. This suggests hyperglycemic preferentially drive iron accumulation in proximal tubules. Conversely, iron dextran administration promotes iron loading in interstitial macrophages—a phenomenon proposed to ameliorate kidney dysfunction in adenine-CKD models [40]. Examining macrophage iron status in THU-treated mice could elucidate their role in THU’s antifibrotic mechanism.

While our findings demonstrate THU’s therapeutic potential, several limitations merit consideration. The 20-week endpoint in our C57BL/6-based DKD model—a strain resistant to diabetic nephropathy [47]—exceeds typical study durations. We intentionally delayed THU intervention until significant kidney injury manifested to assess therapeutic efficacy. Nevertheless, earlier intervention might reveal protective effects during DKD development. Additionally, transcriptomic analysis employed modest sample sizes (*n* = 2/group), potentially limiting statistical power.

In summary, we characterized renal iron redistribution in the DKD model and validated its pathogenic role using HG/PA/TGF-β1-exposed HK-2 cells. The iron ionophore THU effectively mitigated fibrosis both *in vivo* and *in vitro*, primarily by correcting intracellular iron accumulation and the associated oxidative stress-TGF-β axis. These findings suggest THU’s potential for treating diabetic kidney disease by targeting tubular iron dysregulation without necessarily altering systemic iron homeostasis.

## Supplementary Material

Supplemental Material

Supplemental Material

Supplemental Material

Supplemental Material

## Data Availability

RNA sequencing data were deposited in the NCBI Sequence Read Archive (SRA) under BioProject PRJNA1246155. Raw reads for individual samples were accessible *via* the SRA accessions SRR32966409–SRR32966416. The (supplementary tables Table S1 and S2) supporting this study are available in Figshare at: https://figshare.com/s/ed11e6e43661a8229812.

## GLOSSARY

DKD: Diabetic Kidney Disease
ROS: Reactive Oxygen Species
DFO: Deferoxamine
DFP: Deferiprone
CKD: Chronic Kidney Disease
PBS: Phosphate-Buffered Saline
THU: β-Thujaplicin
HK-2: Human Kidney-2 (proximal tubule epithelial cell line)
SV40-MES-13: SV40-transformed Mesangial Cell Line-13
FBS: Fetal Bovine Serum
BUN: Blood Urea Nitrogen
AST: Aspartate Aminotransferase
ALT: Alanine Aminotransferase
H&E: Hematoxylin and Eosin
PAS: Periodic Acid-Schiff
DAB: 3,3′-Diaminobenzidine
ACR: Albumin-to-Creatinine Ratio
DEGs: Differentially Expressed Genes
ICP-MS: Inductively Coupled Plasma Mass Spectrometry
SD: Standard Deviation
