# Supplementary figures and images for: β-Thujaplicin Mobilizes Renal Tubular Iron to Alleviate Diabetic Kidney Disease
Source: Ren Fail. 2026 Apr 29;48(1):2657666. doi: 10.1080/0886022X.2026.2657666 (PMC13134742; doi:10.1080/0886022X.2026.2657666)

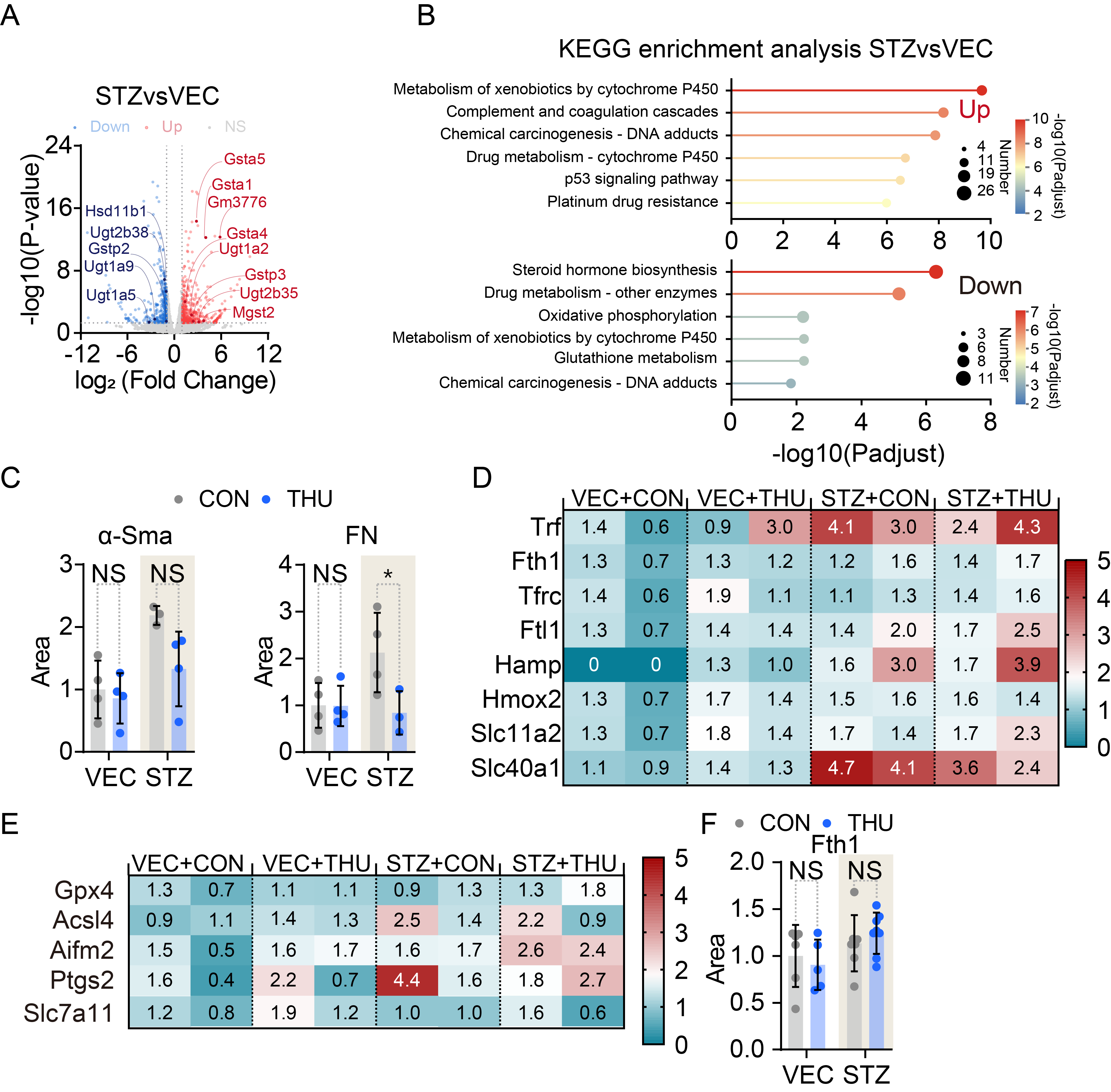

Supplement: Supplemental Material [file IRNF_A_2657666_SM8977.tif]

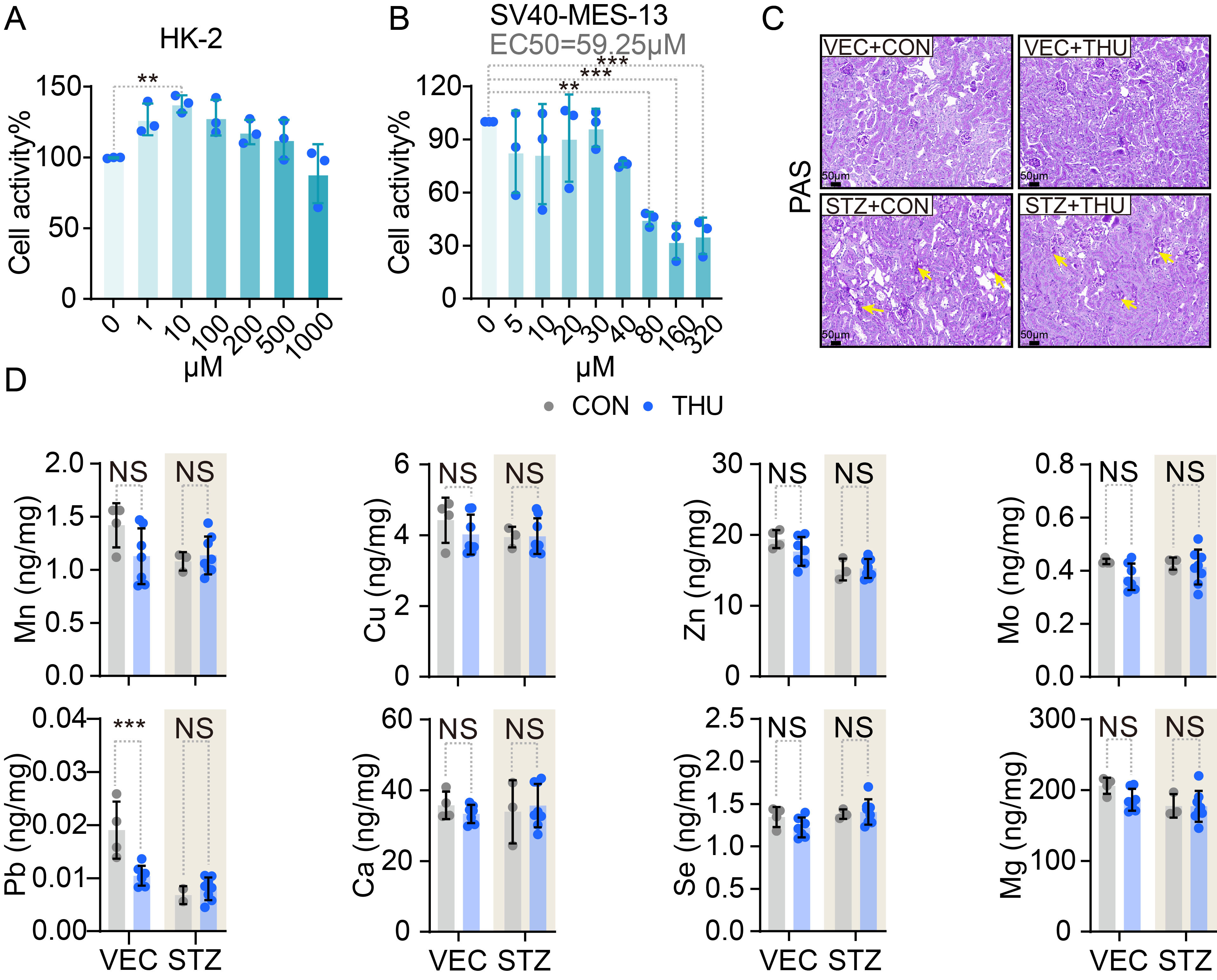

Supplement: Supplemental Material [file IRNF_A_2657666_SM8976.tif]

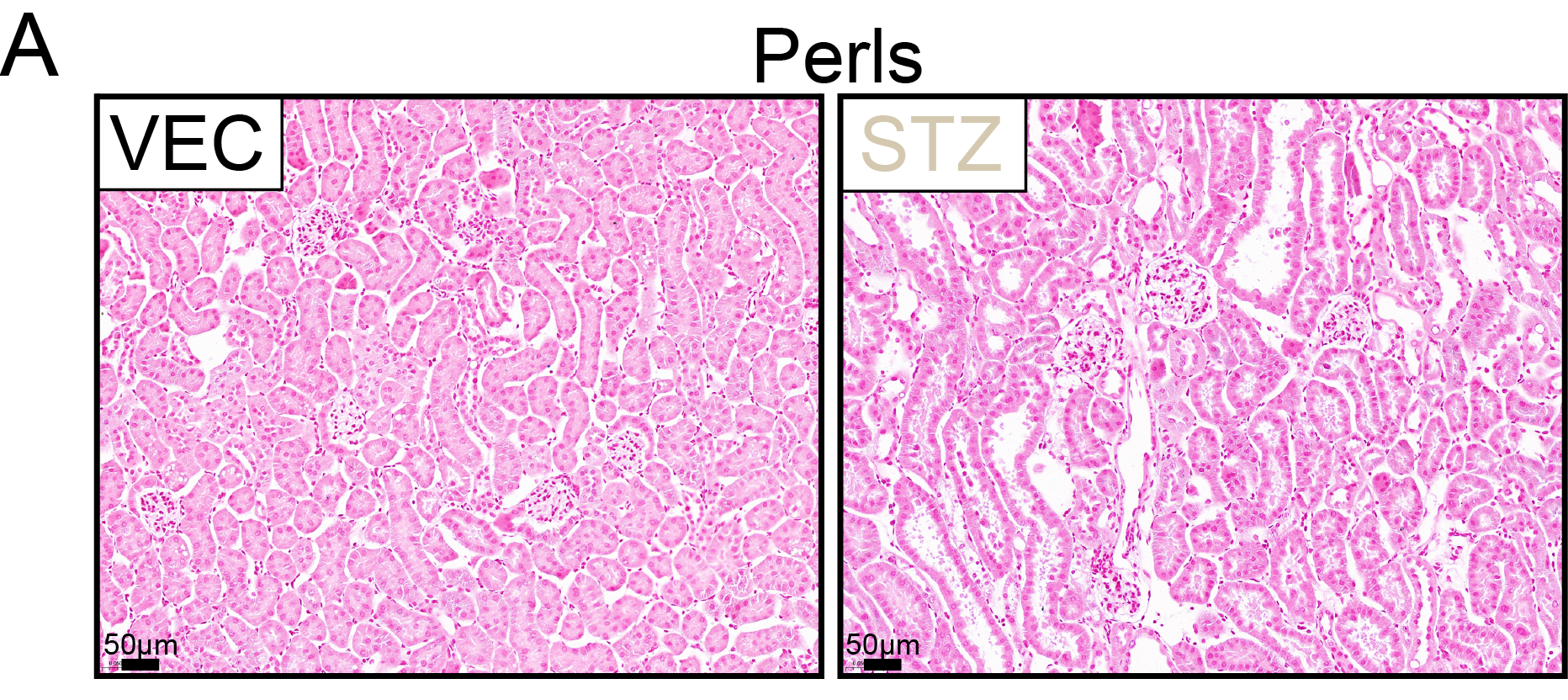

Supplement: Supplemental Material [file IRNF_A_2657666_SM8959.tif]
